# Long-lived water clusters in hydrophobic solvents investigated by standard NMR techniques

**DOI:** 10.1038/s41598-018-36787-1

**Published:** 2019-01-18

**Authors:** Kouki Oka, Toshimichi Shibue, Natsuhiko Sugimura, Yuki Watabe, Bjorn Winther-Jensen, Hiroyuki Nishide

**Affiliations:** 1 Department of Applied Chemistry, Waseda University, 3-4-1 Okubo, Shinjuku, Tokyo 165-8555 Japan; 2 Materials Characterization Central Laboratory, Waseda University, 3-4-1 Okubo, Shinjuku, Tokyo 165-8555 Japan; 3 Department of Advanced Science and Engineering, Waseda University, 3-4-1 Okubo, Shinjuku, Tokyo 165-8555 Japan; 4 Research Institute for Science and Engineering, Waseda University, 3-4-1 Okubo, Shinjuku, Tokyo 165-8555 Japan

## Abstract

Unusual physical characteristics of water can be easier explained and understood if properties of water clusters are revealed. Experimental investigation of water clusters has been reported by highly specialized equipment and/or harsh experimental conditions and has not determined the properties and the formation processes. In the current work, we used standard ^1^H-NMR as a versatile and facile tool to quantitatively investigate water clusters in the liquid phase under ambient conditions. This approach allows collection of data regarding the formation, long lifetime, stability, and physical properties of water clusters, as a cubic octamer in the liquid phase.

## Introduction

The unusual physical characteristics of water, such as its high boiling/freezing point, the abnormal temperature dependency of its density, and ice nucleation for freezing, can be easier explained and understood if water exists as clusters rather than isotropic molecules. This has prompted intensive experimental and theoretical investigation of the structure and properties of water clusters^1–20^. However, experimental studies of water cluster in gas-phase are reported by the need for highly specialized equipment and/or harsh experimental conditions^3,5–7^. While these studies have been successful in detecting water clusters, they have not determined the properties of water clusters or directly and quantitatively measured the processes whereby they form and transform.

Frank *et al*. proposed the first idea that water molecules orient themselves preferentially around the hydrophobic benzene^9,10^. There have been few experimental studies on water clusters in a hydrophobic solvent^11–15^. Lange *et al*. analysed water in benzene by Fourier transform infrared (FT-IR) transmission/absorption and X-ray absorption spectroscopy^11^. Thorsten *et al*. performed IR measurements of water-containing carbon tetrachloride and suggested a cluster-like structure of water^12^. Nakahara and Wakai preliminarily suggested that the ^1^H-NMR signals of water clusters in CCl_4_, C_6_D_6_ and CDCl_3_ shift from those of dissolved and bulk water^15^.

Here, we describe experimental characterization of water clusters by standard NMR spectroscopy under ambient conditions in hydrophobic solvents (benzene is a typical example). A metastable water cluster easily forms by cooling the hydrophobic solvents saturated with water. The samples exhibit an additional NMR signal assigned to water protons with strong hydrogen bonding (“water clusters”) separate from the signals of water dissolved in the hydrophobic solvent (“dissolved water”) and water aggregates (“bulk water”)^15,21^. The ^1^H-NMR signal specifically related to water clusters allows both qualitative and quantitative characterization. In the current work, we found that there was only one type of water clusters to be generated in hydrophobic solvents. We also investigated thermodynamic properties, kinetics, and additional information of water clusters.

## Results and Discussion

**Figure 1:**
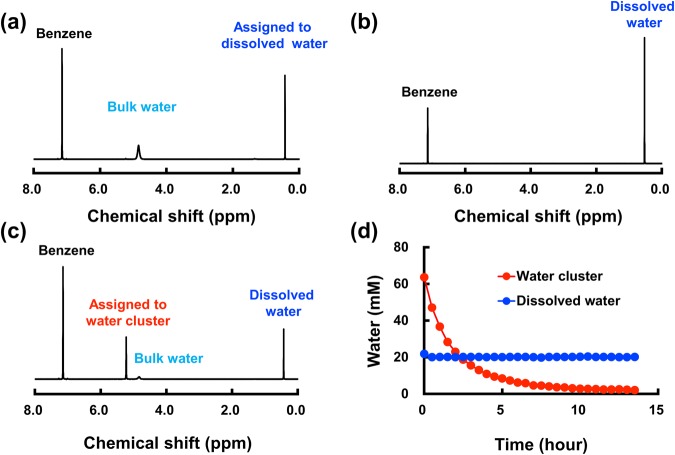
^1^H-NMR spectra of long-lived water clusters. (**a**) ^1^H-NMR spectrum of benzene-d6 mixed with a small amount of water (0.57% water volume content in benzene-d6) at 298 K. (**b)** ^1^H-NMR spectrum of water in benzene-d6 kept at 343 K. (**c)** ^1^H-NMR spectrum of water in benzene-d6 solution cooled to 298 K. (**d)** Changes of the proton signal intensities assigned to the water cluster and dissolved water with time at 283 K. The residual proton signals of benzene in (**a**–**c)** are from residual benzene in the benzene-d6 solvent.

Slightly more water than the solubility limit (0.57% water volume) was added to benzene-d6 in a sealed NMR tube at 298 K, and the tube was then warmed at 343 K to make the homogeneous sample solution fully saturated with dissolved water. The typical ^1^H-NMR spectra before and after warming are shown in Fig. 1a,b, respectively, where the proton signals are assigned to bulk water and dissolved water. ^1^H-NMR chemical shift at a higher magnetic field indicates that dissolved water exists as single molecules^15,21^. The solution was then cooled to 298 K. The ^1^H-NMR spectrum of the supersaturated solution contains an additional sharp signal at significantly lower magnetic field than that of trace bulk water (Fig. 1c). The proton signal with a very large chemical shift of 5.25 ppm suggests the presence of water with a very strong hydrogen-bonding structure, which we call a “water cluster”, compared with that of bulk water^15^. The additional sharp signal with the chemical shift of 5.25 ppm appears also through sonication of the same mixture of benzene-d6 with a small amount of water (Supplementary Fig. S1b). We also measured D_2_O concentration-dependent chemical shift of water clusters in Fig. S1c,d. These results validate that the observed peak is from a water entity. The dissolved water (single molecules) is stable and entropic favourable below the solubility limit. When the dissolved water becomes supersaturated, the single water molecules quickly and spontaneously form water clusters.

It should be noted that the instantaneous appearance of the ^1^H-NMR signal is not observed for water mixtures of polar solvents, where the proton signal of dissolved water continuously shifts downfield with increasing water concentration (Supplementary Fig. S2a). The proton signal assigned to the water cluster is also observed for benzene derivatives, such as toluene and xylene (chemical shifts of 5.19 and 5.17 ppm, respectively, Supplementary Table S1), and even for other hydrophobic solvents, such as chloroform (Supplementary Fig. S2b). Appearance of a new very sharp proton signal of water with a large chemical shift is specific to water supersaturated in hydrophobic solvents. The solvent effect to the ^1^H-NMR signals of water clusters at different solvent ratios are shown in Fig. S2c. This suggests that there is only one type of water cluster structures to be generated in hydrophobic solvents such as benzene, toluene, xylene, chlorobenzene, dichlorobenzene, trichlorobenzene, cyclohexane, carbon tetrachloride and chloroform.

**Figure 2:**
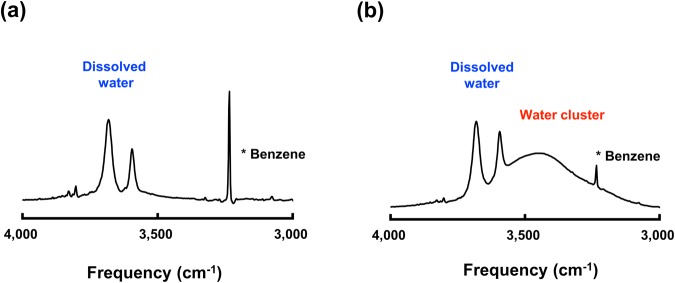
FT-IR spectra of water cluster and dissolved water. (**a**) FT-IR spectrum of benzene-d6 mixed with a small amount of water (corresponding to Fig. 1b) at 298 K. (**b)** FT-IR spectrum of the water cluster and dissolved water in benzene-d6 (corresponding to Fig. 1c) at 298 K. The absorption spectrum of benzene-d6 was subtracted from each measured spectrum. The peaks are assigned to the symmetric (3594 cm^−1^) and asymmetric (3691 cm^−1^) vibration modes of water molecules, strongly tetrahedrally coordinated hydrogen bonding (3100–3400 cm^−1^), and weak hydrogen bonding (3400–3700 cm^−1^)^13^.

As a control experiment, FT-IR spectroscopy was performed for the same sample solutions (Fig. 2). The IR absorption peaks assigned to dissolved water (and bulk water) are overlapped in a broad peak (probably ascribed to a water cluster). For the IR measurements under ambient conditions, it is not surprising that the absorption peak of bulk water (and also the water cluster) broadens because of dynamic hydrogen bonding, which will be discussed later.

Diffusion-ordered and nuclear Overhauser effect (DOSY and NOESY) spectroscopy and spin-lattice *T*_1_ and spin-spin *T*_2_ relaxation time measurement were performed to characterize the water clusters. DOSY spectroscopy gave diffusion coefficients for the protons ascribed to dissolved and bulk water of 5.0 and 2.3 × 10^−9^ m^2^ s^−1^ (Supplementary Fig. S3), respectively, which agree well with previously reported values^22–24^. However, the diffusion coefficient of the water cluster (0.5 × 10^−9^ m^2^ s^−1^) is surprisingly low (about 1/10 and 1/5 that of dissolved and bulk water, respectively). This means that the water molecules in the cluster have very restricted mobility. We showed the picture of the NMR tube (Fig. S1b inset) to show the good homogeneity of the sample. The good homogeneity indicating that the measured effect is not related to artefacts on the glass surface. The *T*_1_ and *T*_2_ measurement gave the correlation time *T*_C_ which characterized the interaction induced by molecular motions, and indicated that the protons in water clusters had a longer correlation time with the nearest neighbour water molecules than that in bulk water (Supplementary Table S2)^14^.

NOESY spectroscopy (Supplementary Fig. S4) shows proton exchange (a negative nuclear Overhauser effect) between the dissolved water and the water cluster, which means that both the dissolved water and the water cluster are solutes in benzene and coexist (are in equilibrium) with a mutual interaction. In addition, the chemical exchange build-up curve by different mixing time indicates that the exchange rate between water clusters and dissolved water is faster than that of the bulk water and dissolved water.

The ^1^H-NMR signal assigned to the water cluster slowly decreases with time (Fig. 1d), although the signal is still detectable after 3 days even at 298 K (Supplementary Fig. S5). The water cluster is in a metastable state, and the data measured within 15 min do not cause a significant difference (error <2%).

The temperature dependences of the NMR signal intensities assigned to dissolved water and the water cluster are shown in Supplementary Fig. S3a. The concentration of the water cluster increases with decreasing temperature accompanied by a quantitative and complementary decrease in the dissolved water amount, and the water cluster concentration reversibly decreases by increasing the temperature.

**Figure 3:**
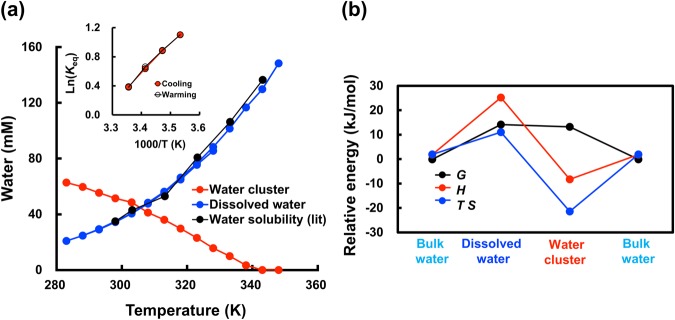
Thermodynamic properties of water clusters. (**a**) Formation of a water cluster by cooling a 0.57% water/benzene-d6 solution from 323 to 283 K (5 K step). The concentrations of dissolved water and the water cluster in benzene-d6 were normalized to the water concentration reported in the literature and handbook^25^ at 298 K. Inset: van’t Hoff plots for the equilibrium between the dissolved water and the water cluster (*K*_eq_: apparent equilibrium constant). (**b)** *H* and *S* values for bulk water at 298 K were cited from ref.^26^ which assumed values of zero at 273 K. The Δ*H* and Δ*S* values between bulk water and dissolved water in benzene were taken from ref.^27^. The Δ*H* and Δ*S* values of the water cluster are from this work. The Δ*G* values were calculated using the classical equation Δ*G* = Δ*H*−*T*Δ*S*.

To investigate the equilibrium between dissolved water and the water cluster, the reversibility, or quasi-thermodynamical stability, of water cluster formation was analysed by the classical van’t Hoff plot (Inset of Fig. 3a). The Δ*H*, Δ*S*, and Δ*G* values for water cluster formation from dissolved water were determined from the straight line in the van’t Hoff plot to give the energy diagram (Fig. 3b). The large enthalpy gains for formation of the water cluster from dissolved water (about 34 kJ/mol) could be the driving force for cluster formation, which can be ascribed to formation of multiple hydrogen bonds. However, cluster formation is accompanied by a large entropy loss (about −110 J/mol K). This can be explained by cluster formation, or formation of a more ordered structure. Δ*G* to form the water cluster from dissolved water, which is the sum of the large enthalpy gain and entropy loss, is slightly negative, and the water cluster is a thermodynamically metastable state.

Transformation from the water cluster to bulk water has a negative Δ*G* value (Fig. 3b), and the water cluster is indeed finally converted to bulk water. An example of decay of the water cluster with time is shown in Fig. 1d. During a separate series of ^1^H-NMR measurements, spinning of the sample tube in the instrument accelerates transformation of the water cluster to bulk water, where second-order kinetics is dominant. The temperature dependency of the decay rate in the Arrhenius plots gives an apparent activation energy of decay of 38 kJ/mol (Supplementary Fig. S6). The large activation energy of cluster decay supports that the water cluster is a metastable state.

**Figure 4:**
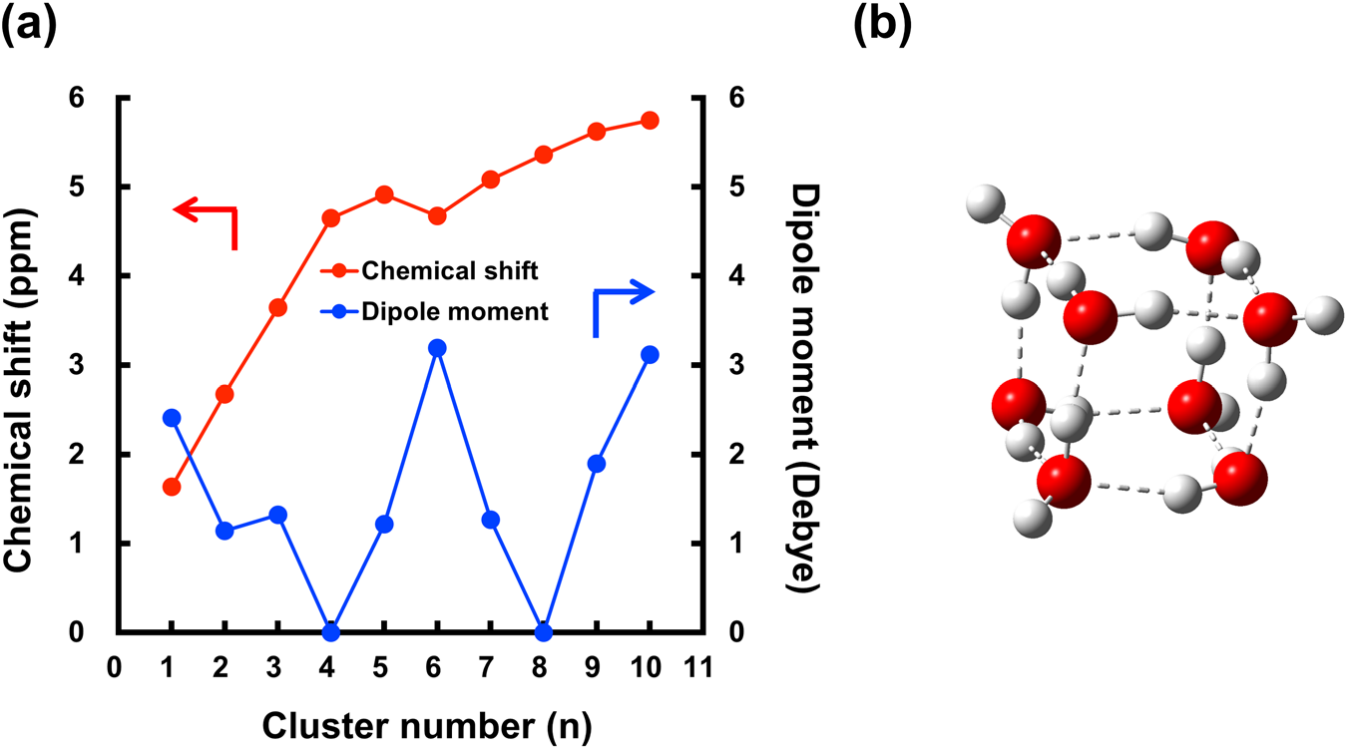
Calculated characteristics of water clusters. (**a**) Calculated chemical shifts (ppm) of the protons and dipole moments (Debye) of water clusters with different *n*. **(b)** Estimated D2d structure of a water octamer. The calculation of NMR chemical shifts and dipole moments were performed utilizing the second-order many-body perturbation theory and gauge-including atomic orbitals with integral equation formalism for the polarizable continuum model of the benzene solvent^29,30^.

The size-specific small water cluster number *n* has been discussed using the experimentally determined continuous shift of the IR frequency with *n* and by ab initio calculations^6,12,28^. Here, we calculated the ^1^H-NMR chemical shifts of the protons of water for different cluster number *n* (Fig. 4a).

The experimentally observed ^1^H-NMR chemical shift of 5.25 ppm (Fig. 1c and Supplementary Table S1) is closest to the predicted chemical shift of 5.36 ppm for the cubic octamer D2d structure in Fig. 4b. This assignment does not conflict with the previous experimental and theoretical studies of cubic octamer^4,5,31^.

The calculation also suggests that the dipole moment of the cubic octamer is zero (Fig. 4a), meaning that the water cluster is a nonpolar entity and differs from “normal” polar water. This helps to explain the long life of the water cluster in hydrophobic solvents. The dipole moment calculation also supports that the observed cluster species is the octamer by excluding polar water clusters with *n* = 5–7, 9, and 10. The suggested cage-like octameric configuration of the water cluster also explains the very low diffusion coefficient of water measured by DOSY spectroscopy, because the diffusivity of the water molecules in the highly ordered water cluster is significantly reduced.

Shields *et al*.^4^ calculated Δ*H* for formation of an octamer with a static stable hydrogen-bonded structure to be −241 kJ/mol, whereas our experimental value is Δ*H* = −34 kJ/mol (Fig. 3b). The difference between the two values suggests that the water cluster formed in this study could be composed of dynamic rather than static hydrogen bonding. This agrees with the experimental ^1^H-NMR signal with a large chemical shift being observed as a sharp peak rather than split peaks owing to individual protons. Dynamic hydrogen bonding in the size-specific cluster (*n* = 8) is one of the features of the thermodynamically metastable water cluster formed in hydrophobic solvents.

Conventional ^1^H-NMR is very effective to investigate the water clusters that easily form in hydrophobic solvents under ambient conditions because of its superior signal resolution to IR and the additional information provided by DOSY, NOESY and relaxation time analysis. A thermodynamic study was performed to characterize the “metastable” state of the water cluster and cluster formation from dissolved water. The dynamic hydrogen bonding in the size-specific cluster is also described, in addition to the nonpolar property of the water cluster. We concluded that water clusters are a cubic octamer, which does not conflict with the previous papers, such as the “square-ice” observed in the hydrophobic conditions recently shown by Algara-Siller, G. *et al*.^32^.

Water clusters have attracted significant interest in many biological and chemical systems, for example, in bioinspired materials and devices where water molecules are in contact with or incorporated in organic hydrophobic materials^33–39^. Investigation of water in organic entities as a matrix by ^1^H-NMR is expected to assist formation and stabilization of water clusters and reveal the unique properties of water clusters.

## Methods

### NMR spectroscopy

One-dimensional and two-dimensional ^1^H-NMR spectroscopy were performed with an AVANCE600 spectrometer (Bruker, Yokohama, Japan). The deuterated solvent (0.70 mL), such as benzene-d6, was injected into a 5 mm NMR tube and then deionized distilled water was added with a micropipette. The non-deuterated solvents, such as toluene, xylene, and chlorobenzene, were injected into a 5 mm diameter NMR tube with a 2 mm diameter inner tube containing cyclohexane-d12, which was used as a magnetic field locking system. Tetramethylsilane was used as the internal reference (0.00 ppm). The standard methods of ^1^H-NMR, NOESY, and DOSY spectroscopy were used^40^. The NOESY experiments were performed with a 200 ms mixing time. The DOSY experiments were performed under a stimulated echo sequence using bipolar gradients and a longitudinal eddy current delay. A diffusion delay of 40 ms and a gradient pulse length of 3 ms were used to obtain appropriate curves (25 points) for inverse Laplace transformation. The water concentration (mol %) was normalized using saturated dissolved water (0.312 mol %) at 298 K^25^.

### IR spectroscopy

The IR spectra were recorded in transmission mode with a Nicolet 6700 FT-IR spectrometer (Thermoscientific). The samples were kept inside a liquid cell with KBr windows and a polytetrafluoroethylene spacer. For each measurement, the absorption spectrum of benzene-d6 was subtracted from the measured spectrum.

### Theoretical calculations

All of the calculations were performed with the Gaussian 09 program^41^. Reported water cluster structures optimized by MP2/CBS-e were used^4^. The theoretical NMR chemical shifts were calculated as the difference of isotropic shielding of the tetramethylsilane at 0.00 ppm. The second-order Møller–Plesset perturbation theory and the gauge-invariant atomic orbital method^29,30,42^ were used combined with the 6–31 + G(d,p) basis set and integral equation formalism for the polarizable continuum model of the benzene solvent.

### Reagents

Commercially available deuterated benzene-d6 (ACROS organics), methanol-d3, dimethyl sulfoxide-d6, acetonitrile-d3, deuterium oxide (Sigma-Aldrich), toluene, xylene, and chlorobenzene (Kanto Kagaku) were used without further purification.

## Electronic supplementary material

Supplementary Information
