# Supplementary material for: Long-lived water clusters in hydrophobic solvents investigated by standard NMR techniques
Source: Sci Rep. 2019 Jan 18;9:223. doi: 10.1038/s41598-018-36787-1 (PMC6338722; doi:10.1038/s41598-018-36787-1)
Supplement: Supplementary file 1 — Supplementary Information [file 41598_2018_36787_MOESM1_ESM.docx]

**Supplementary Information**

**Long-lived water clusters in hydrophobic solvents investigated by standard NMR techniques**

Kouki Oka^1^, Toshimichi Shibue^2^*, Natsuhiko Sugimura^2^, Yuki Watabe^2^, Bjorn Winther-Jensen^3^ and Hiroyuki Nishide^1,4^*

^1^Department of Applied Chemistry, ^2^Materials Characterization Central Laboratory, ^3^Department of Advanced Science and Engineering, and ^4^Research Institute for Science and Engineering, Waseda University, 3-4-1 Okubo Shinjuku, Tokyo 165-8555, Japan


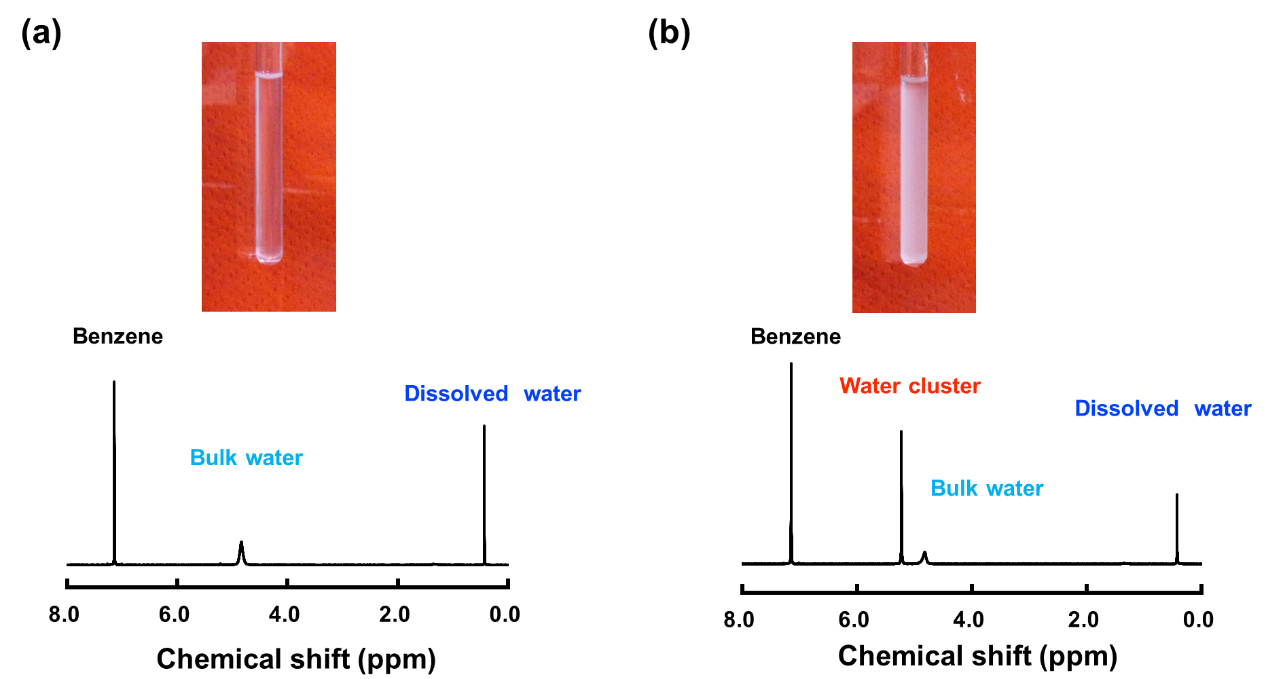


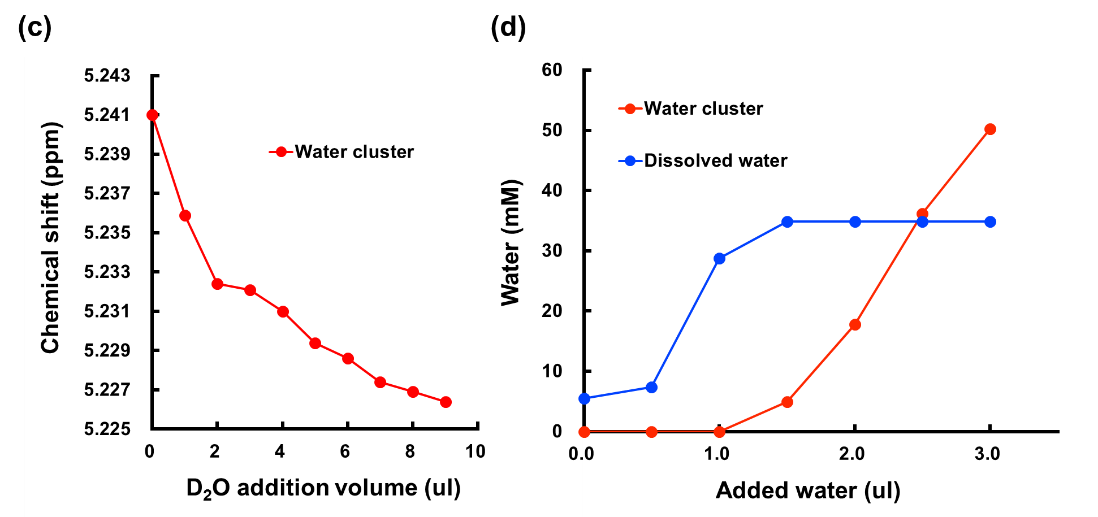


Figure S1. ^1^H-NMR spectrum of the water cluster prepared by sonication.

^1^H-NMR spectrum of benzene-d6 mixed with a small amount of water (0.57% water volume content in benzene-d6) at 298 K (a) before and (b) after sonication of the NMR tube containing the mixture for 1 minute in a sonication bath. Inserts: The simple mixing of bulk water in benzene shows a clear appearance. After sonicated, the sample was a homogeneous cloudy appearance. (c) D_2_O concentration-dependent chemical shift of water clusters (d) Peak intensity (mM) as a function of added water and sonication.


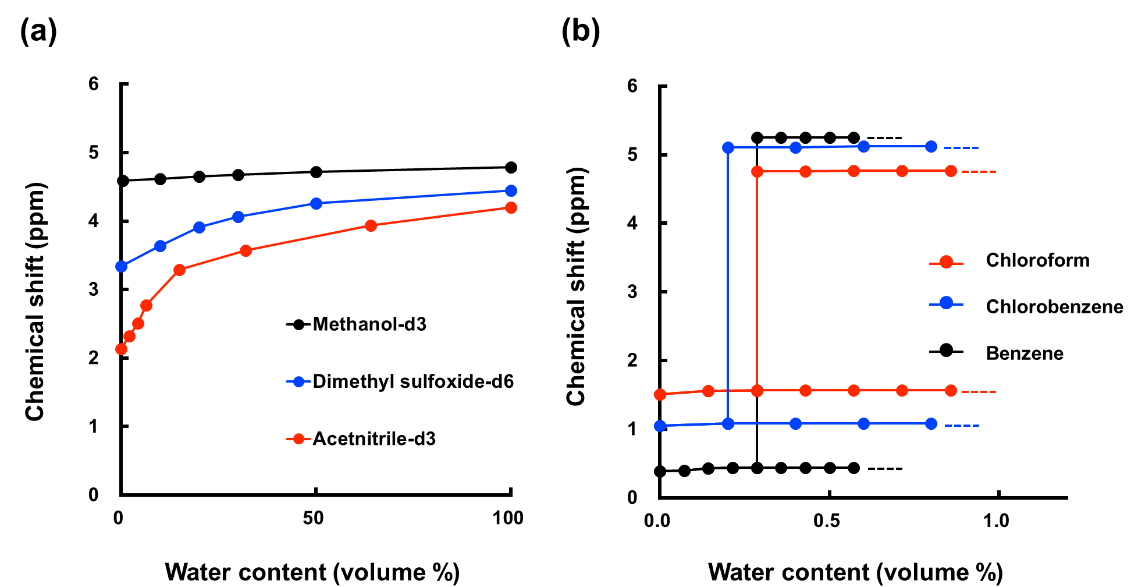


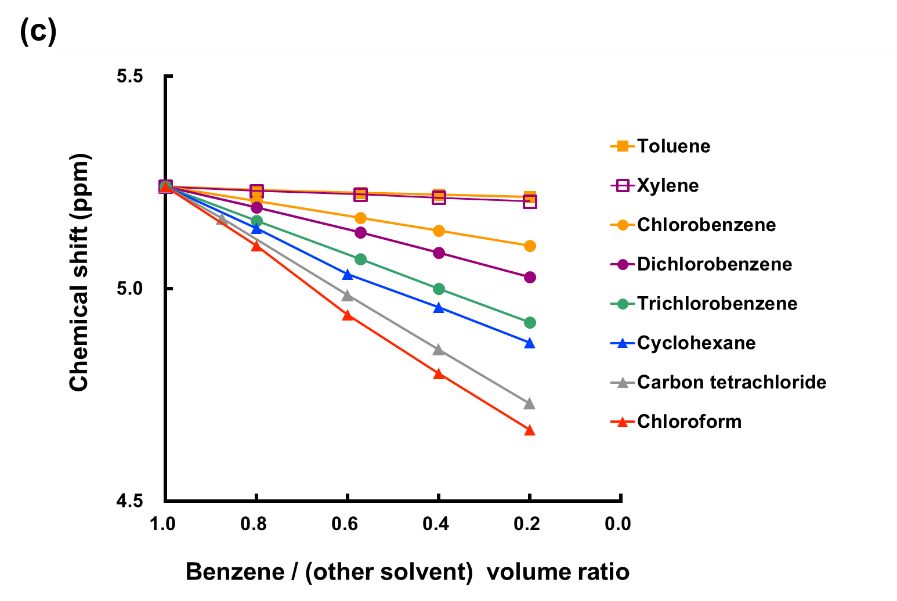


Figure S2. Chemical shifts of water in various solvents.

(a) Chemical shifts of water protons in the polar solvents methanol-d3, dimethyl sulfoxide-d6, and acetonitrile-d3 as a function of the water volume content at 298 K. (b) Chemical shifts of the water protons in benzene-d6, chlorobenzene, and chloroform-d1 as a function of the water volume content at 298 K. (c) Solvent effect of water clusters in mixtures of different hydrophobic solvents at varying ratios. The mixtures with water were prepared through sonication.

There is only one type of water cluster structure to be generated in benzene and other tested hydrophobic solvent. If the structure of the water cluster in benzene and in other solvent were different, the ^1^H-NMR signals would not be shifted in a linear manner at different benzene ratios.


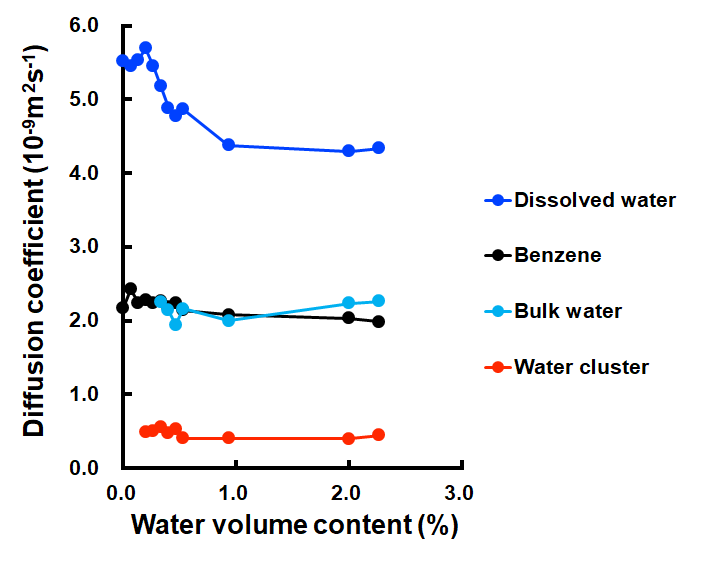


Figure S3. Diffusion coefficients of the water protons determined by DOSY spectroscopy in benzene-d6 solution as a function of the water volume content.

The mixtures with water were prepared through sonication. We observed reproducible of low diffusion coefficient of water cluster with different NMR tubes (hydrophobic surface coating by silane coupling agent) and different magnetic field (500MHz) of NMR


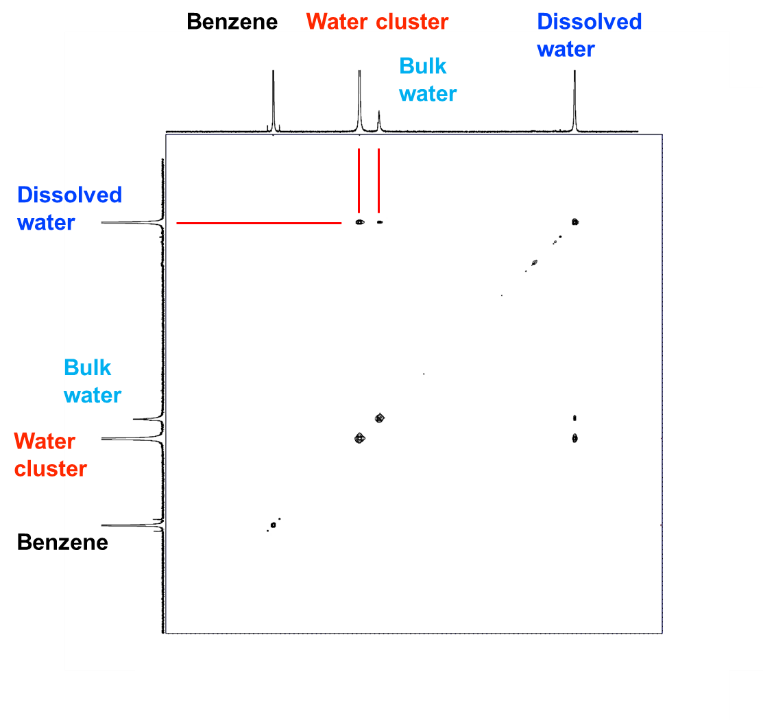

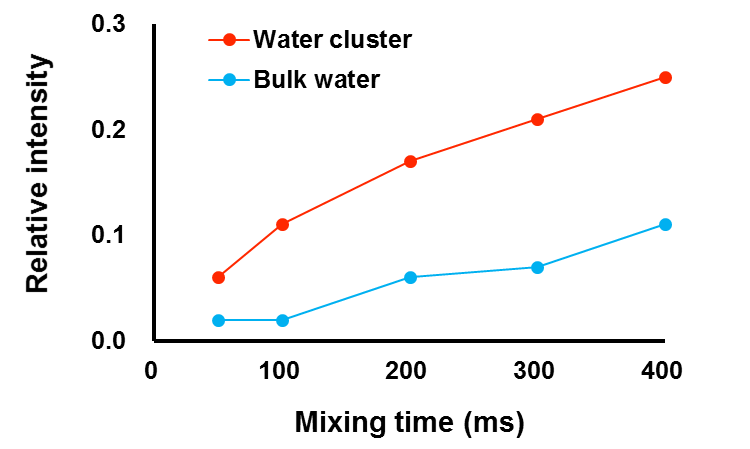


Figure S4. NOESY spectrum and build up curve of water in benzene-d6 (0.57% water volume) at 298 K.


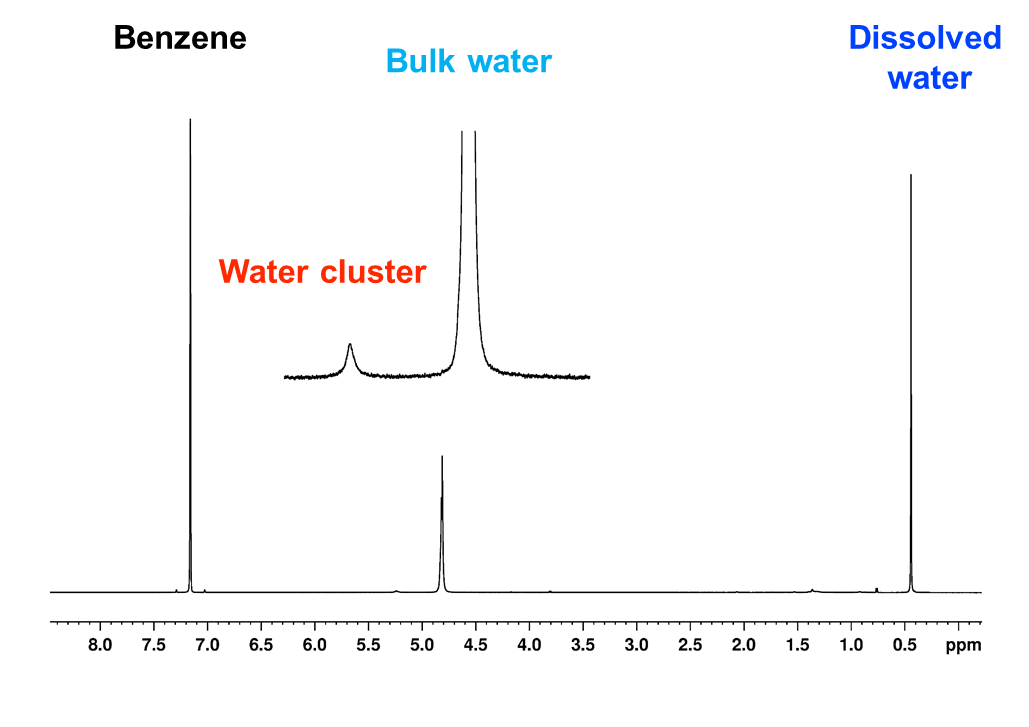


Figure S5. ^1^H-NMR spectrum of long-lived water clusters.

^1^H-NMR spectrum of water in benzene-d6 solution after 3 days standing at 298 K after water cluster formation.

.


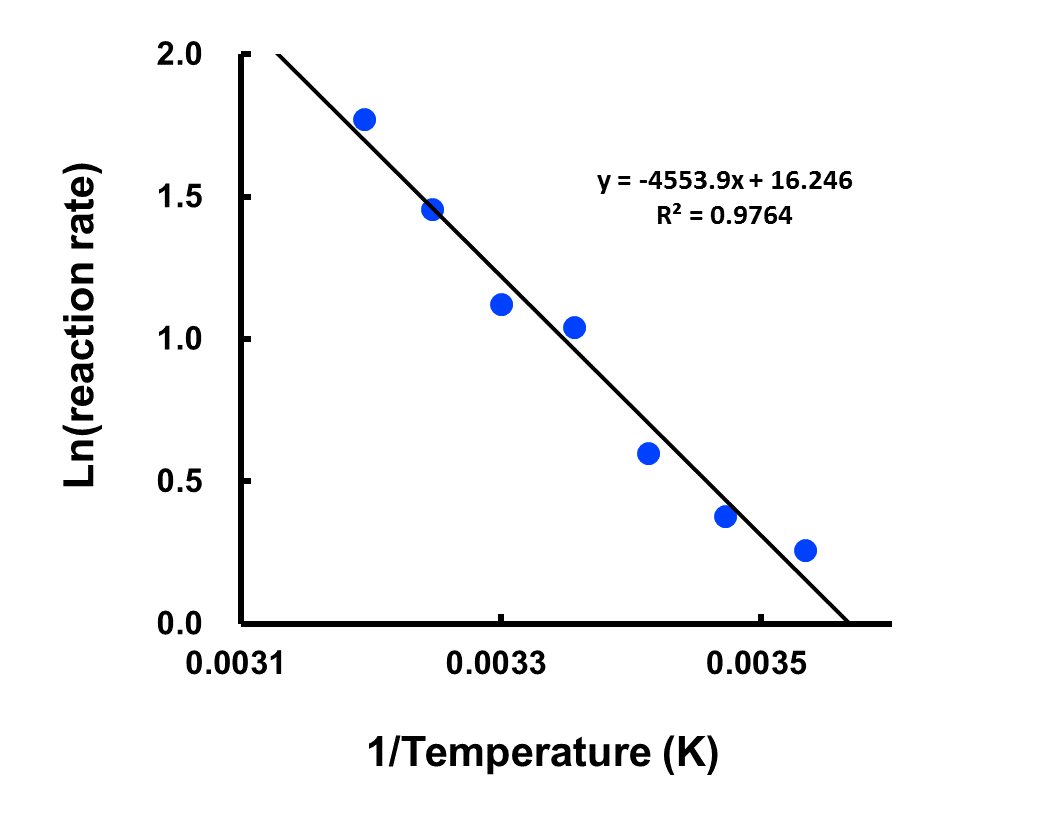


Figure S6. Arrhenius plot.

Arrhenius plot of the decrease of the proton signal intensity ascribed to the water cluster in the temperature range 283–313 K to estimate the activation energy of decay of the water cluster.

**Table S1. Chemical shifts of water in benzene families. Chemical shifts of the protons assigned to the water cluster, bulk water, and dissolved water in benzene and its derivative solvents at 298**


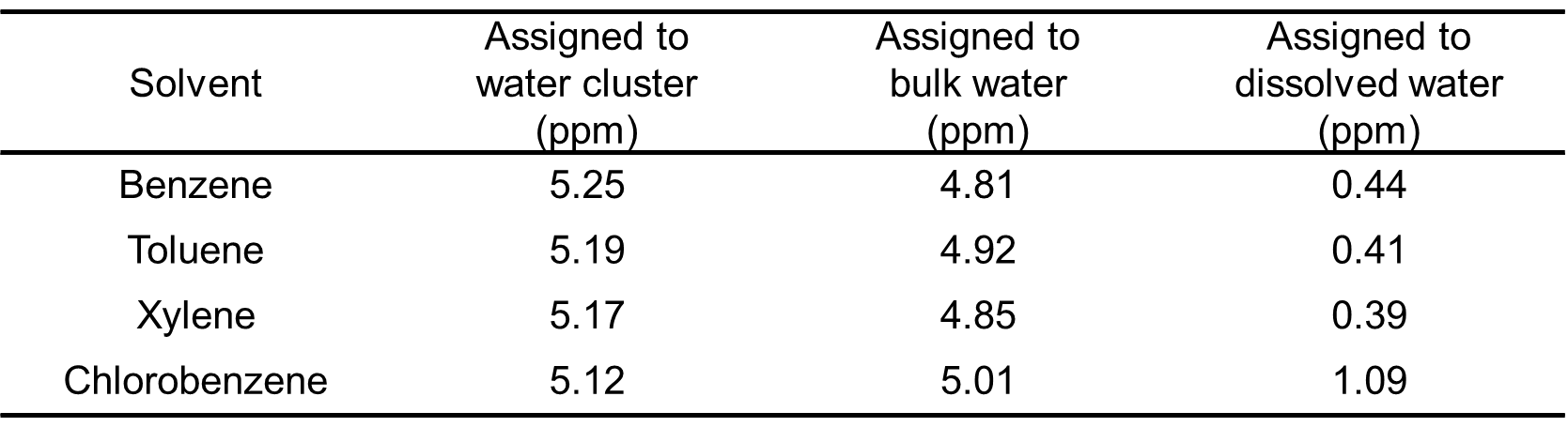


**Table S2. Spin-lattice relaxation times(*T*_1_), spin-spin relaxation times (*T*_2_) and correlation time *T*_C_ at 298 K.**


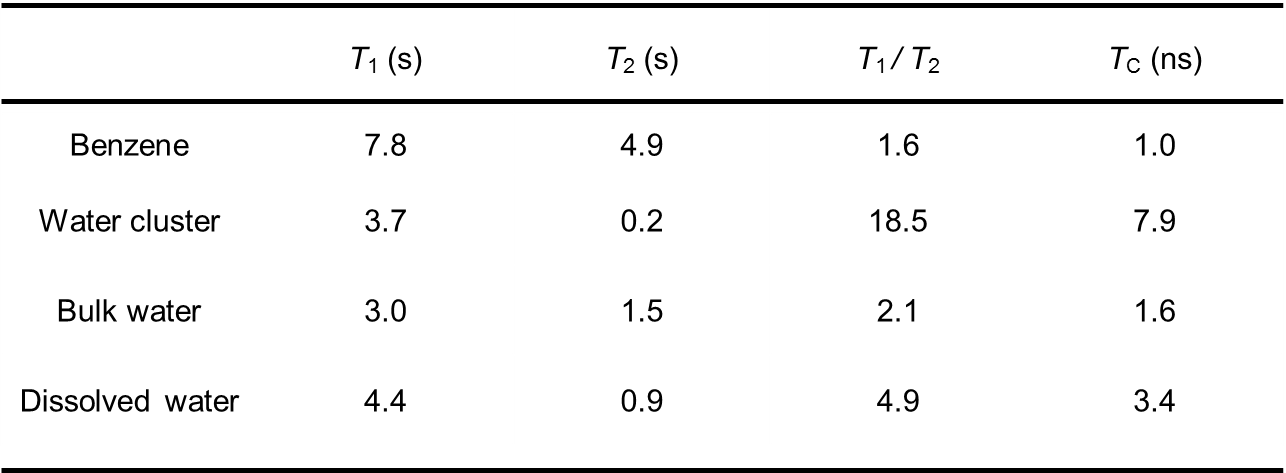


*T*c : correlation time were calculated from ref ^14^.
